# A Stacked Human Activity Recognition Model Based on Parallel Recurrent Network and Time Series Evidence Theory

**DOI:** 10.3390/s20144016

**Published:** 2020-07-19

**Authors:** Peng Zhang, Zhenjiang Zhang, Han-Chieh Chao

**Affiliations:** 1 Department of Electronic and Information Engineering, Key Laboratory of Communication and Information Systems, Beijing Municipal Commission of Education, Beijing Jiaotong University, Beijing 100044, China; zhangpeng11@bjtu.edu.cn; 2 School of Software Engineering, Beijing Jiaotong University, Beijing 100044, China; 3 Department of Electrical Engineering, National Dong Hwa University, Hualien 97401, Taiwan; hcc@mail.ndhu.edu.tw

**Keywords:** human activity recognition, recurrent neural network, time series evidence theory

## Abstract

As the foundation of Posture Analysis, recognizing human activity accurately in real time assists in using machines to intellectualize living condition and monitor health status. In this paper, we focus on recognition based on raw time series data, which are continuously sampled by wearable sensors, and a fine-grained evidence reasoning approach has been proposed to produce a timely and reliable result. First, the basic time unit of input data is selected by finding a tradeoff between accuracy and time cost. Then, the approach uses Long Short Term Memory to extract features and project raw multidimensional data into probability assignments, followed by trainable evidence combination and inference network that reduce uncertainly to improve the classification accuracy. Experiments validate the effectiveness of fine granularity and evidence reasoning while the final results indicate that the recognition accuracy of this approach can reach 96.4% with no additional complexity in training.

## 1. Introduction

Recently, the advent of new technologies in machine learning (ML) together with the extraordinary enhancement of data collection system and computing capability has immensely facilitated human life [1], which draws more attention and resources to physical health. Physical rehabilitation [2], an important component of healthcare, has emerged as a universal service for more than just treatment of the wounded, but also adolescents’ improper habit rectification and elders’ behavior surveillance. Injury healing and coordination recovery are indispensable medical methods to ensure rehabilitation, the former focuses on the therapy depending on the pathology while the latter concentrates on recuperation that minimizes the impact on daily life. Series data are acquired during the recovery course [3], from which ponderable information can be extracted after requisite processing.

Human activity recognition(HAR), which uses machine learning methods to process various categories of data or their combination and recognizes daily activities such as walking and running, is a research hotspot in posture analysis that is featuring in coordination recovery. There are two major recognition methodologies, namely model-driven and data-driven methods [4]. The former classify activities based on handcrafted features extracted by using prior knowledge, which requires expertise and additional computing resources, while the latter acquiring high-level and meaningful features by training an end-to-end neural network [5]. In human activity recognition studies, the majority of data object is focused on static images and videos, and researchers have conceived and implemented many models and algorithms to describe human activity recorded in these formats from diverse perspectives [6,7,8]. However, the existence of following drawbacks or restraints hampers the practical application of static image- or video-based recognition methods [3], (1) Restricted capturing scope. On account of objects’ movements and low camera coverage in remote and sensitive areas, the fixed acquisition equipments cannot provide uninterrupted service, moreover it will cost tremendous amounts of time and money to extend the acquisition network; (2) Redundant information. The behavior related pixels only occupy part of data, meanwhile, many same objects have been repeatedly shot with time. (3) Large demand of computing resource. Since image and video data are elusive for computers, finding out related positions and categorizing involved actions require massive calculations and analyzes that are not suitable for time-sensitive applications. However, static images or videos are beneficial in the labelling or verification procedure for its convenience to direct viewing.

During the last decade, flourishing development of sensor and computer technology prospers the wearable devices that acquire data conveniently, enabling activity recognition to be ubiquitous. Additionally, the recognition problem can be reformulated into a classification problem based on time series analysis for numerical data which is much more concise. The classification model contains three essential processes, data preprocessing that filters unnecessary information based on physical characteristics of sensors and objects together with the statistical property of data, classifier that selects the maximum possible category based on extracted features, and data translation that can be divided into two sub-procedure, data conversion and data projection.

Data conversion is an intermediate process between data preprocessing and data projection, the purpose of which is intended to convert preprocessed data into input data that are facile to be classified with effective remained information. Since the massive preprocessed data generally have correlation in spatial or temporal domains, special methods have been proposed to constitute basic data units that provides convenience for feature extraction. For instance, some proposal models for images and videos and some speech signal modeling methods are conducive to object detection and speech recognition.

Data projection, the last process before classification, which maps basic data units into special space, describes input data from various perspectives and eventually translates the descriptions into features containing sufficient discrimination. This procedure is generally integrated with classifiers or regulated by preset rules to learn from the input data with the purpose of improving the accuracy.

Traditional time series analysis methods generally classify or recognize targets based on data during a consecutive period of time that is regular and periodic. Although the time domain data are translated into diverse of representations in different methods, an invariable precondition that the data merely contains an unabridged object has been assumed to ensure the accuracy. It has been confirmed that this precondition is effective in much research [9,10]; however, to detect the duration of an object is cumbersome and consumes large amounts of computing resources. Therefore, a simplified suboptimal scheme that selects an constant length window based on statistics to transform data into subsequences regarded as quasi-objects has been applied. For human activity recognition, owing to the continuous repetition and fluctuation of activities, the appropriate constant parameter is scarcely to be sought to satisfy the precondition. As the first step of the whole model, the length of window directly affects the real-time performance and the information being contained. Moreover, with the development of Edge Computing [11,12,13], parallelization and expansibility will be the new indicators of models designed to solve practical problems.

In this paper, inspired by the research in Automatic Speech Recognition (ASR) [14,15], raw data replaces handcrafted features as the input of the model to avoid resource consumption and deviation caused by potential unnecessary or redundant handcrafted features. To monitor the details of kinestate and reduce redundancy of input data, we propose a stacked human activity recognition model that mainly includes several mapping subnetworks and a combination network. First, a fixed proper interval for each input is sought out based on the tradeoff between accuracy and promptness. Then, several parallel mapping subnetworks based on Long Short Term Memory(LSTM) are used to learn features from the input owing to its memory ability and gates which is appropriate to the motion data analysis. Finally, the results of all subnetworks are fused by an improved evidence combination network based on the characteristic of classification problems to integrate the final recognition results. Compared to the existing recognition methods, the main contributions of this article are summarized as follows: (1) directly training neural network with the fine-grained raw data instead of intermediate feature representation to streamline algorithm structure and provide meticulous activity detection and recognition; (2) using parallel subnetworks instead of one huge network to implement data translation; (3) fusing probability assignments of different subnetworks with a new Dempster-Shafer evidence theory-based algorithm to enhance discrimination ability.

The remainder of the paper is organized as follows. Related works are reviewed in Section 2; Section 3 discusses the constant length of input mentioned before; in Section 4, we present the architecture of the recognition model; Section 5 concerns the experiment simulation and analysis of results; finally, Section 6 draws some conclusions.

## 2. Related Work

The earliest research of human activity recognition was traced back to 1970s when Johansson provided a visual model to perceive human motion [16]. Since the 1990s, researchers attempted to involve human activities in neoteric health-care and entertainment applications based on the flourishing computer vision and data processing technologies [17,18] that brought an extraordinary opportunity for the development of HAR. Two strategies diverged in this study, one is to construct motion models based on data acquired by optical sensors [8,19], including videos and still images. Trajectory-based model [20] and shape-based model [21] are the most prominent methods in this strategy; the former extracts features based on trajectories of key points while the latter estimates human poses to represent human activities. In recent years, convolutional neural network has been the universal algorithm of computer vision on account of its reliability and expeditiousness in the extraction of image features [22], the majority of activity recognition models are based on this efficient algorithm [19]. The other strategy that analyzes fixed wearable sensors data to discover the distinction among various activities is what we implement in this paper. In the literature, practical recognition model is the research hotspot that gives consideration to both theoretical research and practical applications.

Even though the final purpose is to recognize activities, the models are diverse owing to various concepts and scenarios. As for motion data acquisition, some researchers attempted to collect data as comprehensive as possible. Bao and Intille [23] placed multiple biaxial accelerometers on different parts of the body while Yin [24] added extra sensors to record related ambient conditions simultaneously, such as temperature and sounds. Besides, as a typical physiological index to react the intensity and volume, the effectiveness of heart rate has been discussed in [25]. Whereas convenience was taken into consideration, a few sensors were placed in representative parts to gather enough information. He [26] put a single tri-axial accelerometer in clothes pocket, waist bell and trousers pocket separately to enhance the robustness, Kwapisz [27] and Anguita [28] merely used the inner accelerometer of cell-phones that were carried in the front pants pocket and waist respectively. As for data preprocessing, high-frequency noise and low-frequency gravitational components were filtered by technical low-pass and high-pass filters based on frequency statistics of constituents [28,29]. Additionally, the length of time window ranged from 0.08 s to 30 s [3] and the overlapping size of adjacent windows were generally 0 [30] or 50% [31] to regulate the amount and smoothness in subsequence generation.

Data translation, an indispensable module of classification model that translates raw data into special representation, can be regarded as the process of feature extraction. Two principle approaches have been designed to obtain proper features. Statistics related and experience-based methods share the same approach that features are calculated according to assigned rules that have been established in other research, with a distinctive attribute that those features generally have definite meanings, for example, fast Fourier transform(FFT) coefficients that contain the frequency domain information [32], statistics features that describe the distribution characteristics, such as mean and standard deviation [28]. Moreover, special values are regarded as additional features to perfect the description of time domain information, typical examples include the maximum and minimum value together with the corresponding indexes [28]. Some particular rules were proved to be effective in extracting discriminative features, for instance, Cai [29] calculated the maximum distance between time sequence value and a defined base area. Since excessive features cause expensive resource consumption or even beyond the computing capability, dimensionality reduction algorithms have been used to construct lower dimensional space that retain sufficient information while the initial feature is in high dimensional space. The common methods include manifold learning [33] and linear learning, such as principal component analysis (PCA) [34], linear discriminant analysis (LDA) [35] and locally linear embedding (LLE) [36], nonnegative matrix factorization (NMF) [37]. Based on LDA and LE, Tao [33] proposed the ensemble manifold rank preserving algorithm (EMRP) which preserved the ranking order information of intra-class and used the linear combination of multiple alignment matrices to approximate the intrinsic manifold. Furthermore, Guo [38] extracted bidirectional features from time series data and directly processed the tensor-based features with the tensor manifold discriminant projections algorithm (TMDP).

The other approach is to learn features automatically with different network models. Due to the nonlinearity of activation function, raw data are translated into features or even the probability of each category [39,40]. Compared to the aforementioned approach, neither final features nor intermediate outputs of hidden layers in most network models have definite meanings, instead by sketchy concepts. In [41], deep neural network (DNN) that imitate the frame of human brain was used to learn features from input data. Convolutional neural network (CNN)-based classification model was proposed by Ronao [42] to exploit the temporally local dependency of time-series signals while recurrent neural network (RNN)-based models extract features from the sequential input data with the inner memory unit [43]. Ordóñez and Roggen [44] combined the CNN and the LSTM to construct a DeepConvLSTM framework for activity recognition that took into account both local dependency and sequential correlation. Autoencoder was used in [45] to learn latent low dimensional representation of input data from which data were recovered with low reconstruction deviation.

Moreover, the combination of data is an extra problem when it comes to multiple sensors data processing. A common method is to assume that the data acquired by different sensors is independent and regard them as separate classification criterion [25]. Other researchers adopted diverse methods. For instance, Tao [33] selected the representative frequency features of acceleration along each axis respectively and combined them into a vector. Cai [29] treats three axes data of tri-axial accelerometer as an integration, classifying activities based on the resultant acceleration. While data acquired by different sensors or in different axes were treated equally and were synchronously entered into models in neural network-based methods [42].

Learning methods that relate features with corresponding classification results are generally divided into generative methods and discriminative methods. Generative methods calculate the probability of each activity by estimating the joint probability distribution of input and associated classes and select the most possible activity as the final result [46]. Representative algorithms include Bayes network [47] and Hidden Markov Model [29]. Discriminative methods directly estimate the posterior distribution or the decision function to discriminate different activities [46]. Representative algorithms include softmax classifier [42], k-nearest neighbor(kNN) [33] and support vector machine(SVM) [48]. Neural networks can also be treated as discriminative methods when their output is the final result [39].

## 3. Stacked Human Activity Recognition Model

In essence, human activity recognition is a multi-classification problem and the result depends to a great extent on the definition and label of activities. On account of the continuity and similarity of daily activities, some activities are confusable when it comes to the wearable sensor data processed by machine learning algorithms. In fact, it is comprehensible that detailed and comprehensive description is advantageous to classification. However, wearable sensor based activity recognition which synthesizes data acquired by one or more sensors that reflect local actions aims to use the fewest sensors and least fixed point, satisfying the requirement of mobility and portability. Therefore, some mutually exclusive activities turn out to be partly confused from the perspective of numerical data.

Instead of deploying one huge and deep neural network to classify multi-activities, in this paper, we treat each activity as an independent event and arrange several small neural networks to calculate the probability of activities in parallel, following by a combination network to synthesize probabilities into the final result based on evidence combination rule. Considering that human activities are durative and comprise sequence of changes with time, we adopt LSTM to extract features from time series data. LSTM is an improved variation of recurrent neural network. In comparison to the traditional recurrent neural network which appends a parameter to record historical information and represent latent state of neurons, LSTM introduces gates to filter information and separates neurons output from cell state to solve the vanishing/exploding gradient problem [49].

**Figure 1:**
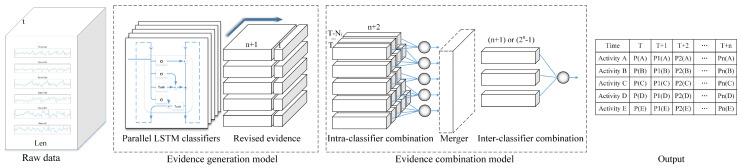
The stacked human activity recognition model.

As shown in Figure 1, the stacked human activity recognition model proposed in this paper has two main sub-models, evidence generation model and evidence combination model. The former extracts features from the raw data and generate original evidence which will be revised based on the common evaluation indicators of classifiers while the latter combines evidence with a two-layer evidence combination network that synthesizes the time domain historical information and multi-classifiers results respectively.

### 3.1. Basic Time Unit of Input Data

As mentioned before, the input data will directly affect the classification results. In wearable sensor-based human activity recognition studies, the majority of time window length selection is inclined to include a complete activity which contain the most information of activities that will conduce to the recognition. However, to find the time interval exactly including one activity from the time series data is another problem waiting to be solved. In practical application, the length of window is usually fixed to facilitate the model constructing. Thus, the frequency of each activity in one window will be variable and possibly not an integer or even less than 1, depending on the length chosen by researchers based on their experiences and experimentations. In this paper, we assume that any subinterval of simple repetitive activities, such as walking and waving, contains enough information for recognition even though those subintervals represent different parts of activities, and complex activities can be regarded as the sequential combination of multiple simple activities. Hence, we attempt to use small window that just include part of the whole activity to classify activities, and the length of window cannot be too short to ensure that the information contained in the interval is enough.

**Figure 2:**
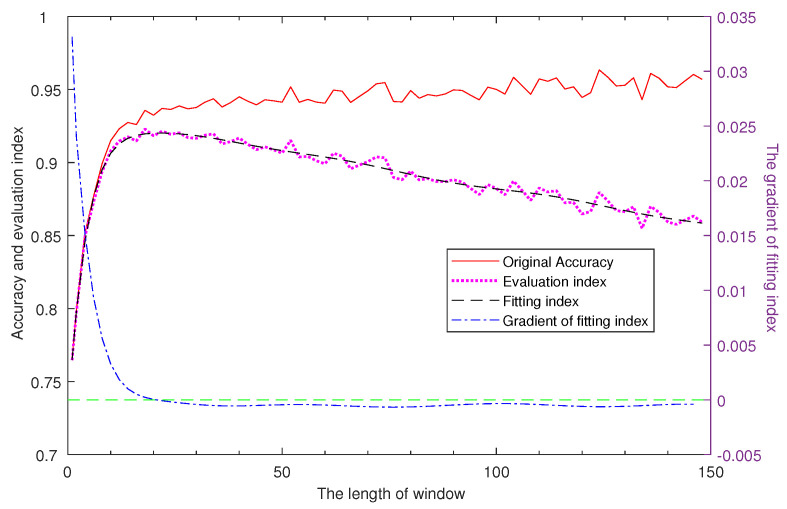
Analysis of the real time strategy. There are four curves in this figure, respectively corresponding to (―) original Accuracy, the accuracy of the two-layer LSTM network where the window length range from 1 to 150; (▪ ▪ ▪ ▪) evaluation index, the index designed to select the length of window; (– – –) fitting index, a thirteen-order polynomial used to fit the evaluation index function; (– - – - –) gradient of fitting index, the gradient of the fitting index. The first three curves refer to the left *Y*-axis while the last curve takes the right *Y*-axis as reference.

We use a two-layer LSTM network of fixed number of nodes to analyzes the accuracy changing with the window length on UCI-HAR data-set [50], and there is a 50% overlapping between adjacent two windows. As shown in Figure 2, the accuracy is increasing with the window length while the growth rate is decreasing, and the accuracy has small range of variation starting from a small window length, thus, we can select an appropriate small window by finding a tradeoff between accuracy and time cost.

On account of that the sampling frequency is much less than the calculating frequency, time cost of recognition model is approximate to the time interval of window in the analysis of the real time strategy. To find the appropriate window length, we present an evaluation index *I* which is increasing with the accuracy *Acc* while decreasing with the window length *Len*, and it is expressed as Equation (1),

(1) $$I=\frac{Acc}{1+Len/Con}$$

where *Con* is a constant that is much larger than the maximum value of window length to avoid the influence caused by the exponential growth of window length at the beginning. We use a multi-order polynomial to fit the trend of evaluation index, and the maximum of index can be found based on the its gradient. As indicated in Figure 2, the index first increased and then decreased, and the fitting index reached its maximum value when the window length is 20 while the corresponding accuracy is 93.24%. In summary, the accuracy reached a high value with a window only including a time interval of 0.4 s (with a sampling frequency of 50 Hz and a 50% overlap), which proved the feasibility and effectiveness of small window in particular scenario that may be promotive in the real-time strategy research. Additionally, the experiments are based on the small window strategy in the rest of this article.

### 3.2. Parallel LSTM Evidence Generation Model

As mentioned above, each activity is regarded as one individual event and we use a parallel LSTM network to calculate the probabilities of activities called Parallel LSTM evidence generation model(P-LSTM-E). This model consists of multiple discriminant modules that process the same input data simultaneously. Each module contains a LSTM network to extract features and a softmax classifier to discriminate classes that has irrelevant parameters, such as the size of LSTM network which directly influence the generation ability. Compared to one huge LSTM network, this model has shorter time consuming for its parallel-processing architecture. Furthermore, there is a significant linear correlation between the total delay caused by calculation and the delay ascribed to the largest branch LSTM network, majority time of which is occupied by model implementation. Generally, multiple classifiers structure increases the number of neurons and computation complexity. The P-LSTM-E model adopts an adaptive strategy that each module select the minimum number of neurons separately to reduce the network size. Moreover, sparse connections within the model decrease the number of weights and biases among neurons. Thus, the computation complexity and requirement of memory resources are restricted to an approximate range around the huge LSTM network.

On account of the discreteness of activities, we use one-hot encoding to label them to define the distances between activities equally. To accommodate multiple classifiers, a two-dimensional coding strategy is applied to replace the traditional fixed coding method. For each activity *a*${}_{i}$, where *i* is a positive integer between 1 and n while n is the number of activities, a vector of length n (*z*${}_{1}$, *z*${}_{2}$, *z*${}_{j}$, …, *z*${}_{n}$) is used to represent the label, the element *z*${}_{j}$ has the Boolean value 1 if and only if i=j while the others are the Boolean value 0. For each classifier *s*${}_{i}$ corresponding to the activity *a*${}_{i}$, all the other activities are merged into a complementary set ${∁}_{A}a_{i}$ to focus on the discrimination of target activity while *A* denotes the universal set of activities. An additional element *z*${}_{n}+1$ is appended to the vector and it has the Boolean value 1 when i≠j while the other elements are the Boolean value 0. Thus, each activity has multi-encodings and in a sense, a matrix of n×(n+1) is applied to label them. The softmax classifiers are used to match the features with multi-column labels and output vectors of normalized possibilities. The objective function is to minimize the cross entropy loss between the possibilities and labels which can train the model to approximate the ground truth. The possibility vectors indicate the certainly of specific activity and the uncertainly of the others respectively that can be regarded as evidence revealed from the raw data by the model.

The n×(n+1) matrix ***E***${}^{t}$ outputted by the evidence generation model denotes the discrimination of input vector ***X***${}^{t}$ during the time interval *t* while the n+1 dimensional vector ***P***${}_{s_{i}}$ is the effectiveness index corresponding to the classifier *s*${}_{i}$. ***E***${}^{t}$ represents the judgement of multiple classifiers to input vectors during one specific time interval; however, missing alarm and false alarm are hard to avoid, it is necessary to modify the discrimination probability matrix based on the statistics characteristics. There are four results according to the comparison between judgement and the ground truth.

True Positive, the judgement is that input data represent the activity which the classifier designed to ascertain, and it is true according to the ground truth. False Positive, the judgement is the same with TP, but it is contrary to the ground truth. True Negative, the judgement is that input data represent activities with the exception of the targeted activity, and it is true according to the ground truth. False Negative, the judgement is the same with TN, but it is contrary to the ground truth.

Moreover, TP, FP, TN, and FN are used to denote the number of inputs belonging to the four results respectively. Among which, TP, FP, and FN are directly associated to the reliability coefficient of probability assigned to targeted activity while FP, TN, and FN are related to the reliability coefficient corresponding to the other activities. These two coefficients are the *i*th and (n+1)th component of ***P***${}_{s_{i}}$ and are respectively calculated as Equations (2) and (3).

(2) $$P_{s_{i}a_{i}}=\frac{TP}{TP+FP+FN}$$

(3) $$P_{s_{i}a_{n}+1}=\frac{TN}{TN+FN+FP}$$

where TP+FP+FN is the total number of inputs that can be or should be judged as the targeted activity and TN+FN+FP is the homologous value to the complementary set of targeted activity. The increasing of TP/TN and decreasing of FP or FN lead to the increasing of ***P***${}_{s_{i}a_{i}}$/***P***${}_{s_{i}a_{n}+1}$ from minimum 0 to maximum 1 which satisfies basic properties of reliability coefficient. The value of other components of ***P***${}_{s_{i}}$ are set to 0 for the reason that the value of the corresponding components of ***E***${}_{s_{i}}^{t}$ are close to 0 and classifiers pay no attention to the categories those components related to. After the adjustment, the sum of components of vector ***P***${}_{s_{i}}$ is less than 1 and the remaining probability is assigned to a new component $\Omega_{i}^{t}$ that denotes the uncertainty of the judgement which means every activity category is possible. Accordingly, a revised evidence matrix $\tilde{E^{t}}$ is generated.

(4) $${\tilde{E}}^{t}=E^{t}⊙P+\Omega^{t}$$

Equation (4) shows the generation of $\tilde{E^{t}}$, where ***E***${}^{t}$ is the initial evidence matrix, ***P*** is the reliability coefficient and the n×1 matrix $\Omega^{t}$ is the new component. ⊙ represents the Hadamard product and + denotes a concatenation of two matrixes that have the same amount of rows. $\Omega_{i}^{t}$ related to classifier *s*${}_{i}$ can be written as Equation (5).  

(5) $$\Omega_{i}^{t}=\sum_{j=1}^{n+1}E_{s_{i}a_{j}}^{t}\left(1-P_{s_{i}a_{j}}\right)$$

where the activity *a*${}_{n}+1$ refers to the additional element ${∁}_{A}a_{i}$.

The evidence matrix $\tilde{E^{t}}$ consists of a series of final judgements from classifiers, each of which can be translated into a Basic Probability Assignment(BPA) in evidence theory with the simple support evidence algorithm that satisfies the following constraints: (1) All the elements are non-negative and the value of empty set *⌀* is 0; (2) All elements add up to a constant value 1. One thing deserves to be mentioned is that the additional element ${∁}_{A}a_{i}$ corresponding to diverse activities are different. Thus, many vacancies should be preset for the new elements generated by the intersection of existing elements in the combination procedure. Moreover, the combination of all the final judgement components will extend the dimension of output to $2^{n}-1$, which result in exponential explosion of weights and computation complexity. Fortunately, the value assigned to additional elements ${∁}_{A}a_{i}$ cannot intersect with the value assigned to activity *a*${}_{i}$, therefore, the (n+1)th element can be merged into the (n+2)th element in each final judgements that will overcome the explosion problem.

Unlike the common method that choosing the class which has the maximum probability among all final judgements as the result, in this paper we use modified Dempster’s evidence combination rule to fuse those evidence with trainable weights. The modified rule still follows the basic combination rule that the combined probability of an element is the sum of all product of basic probability whose intersection is the object element. Each product involves all BPA and the combined probabilities need to be normalized to ensure the probability assigned to empty set is 0. The basic combination rule can be expressed as Equation (6).

(6) $$m_{D}\left(B\right)=\left[m_{1}⊕m_{2}⊕\cdots ⊕m_{n}\right]\left(B\right)=\frac{\sum_{\overset{n}{\underset{i=1}{\cap }}B_{i}=B}\prod_{i=1}^{n}m_{i}\left(B_{i}\right)}{1-\sum_{\overset{n}{\underset{i=1}{\cap }}B_{i}=⌀}\prod_{i=1}^{n}m_{i}\left(B_{i}\right)}$$

where *B*, *B*${}_{i}$ are non-empty elements of BPA and *m*${}_{i}$ denotes different BPAs while *m*${}_{D}$ denotes the combined BPA. Reciprocal of the denominator is the normalization factor which means reallocation of empty intersection probability.

### 3.3. Time Series Evidence Combination Rule

That evidence is circumscribed and a combination rule is requisite to draw the final conclusion. The time series evidence combination rule proposed in this paper adjusts the evidence by considering the effectiveness of classifiers and historical data in short time, after which the evidence is synthesized into one probability vector based on evidence theory and we select the most possible category as the final result. The Dempster’s evidence combination rule is associative and commutative, hence the combination of multiple BPAs can be accomplished by multiple combination of two BPAs and the combination order has no effect on the final result.

According to Equation (6), elements assigned with large probability in respective BPAs will remain large in the combined BPA when it comes to the combination of conflicting evidence while the combination of supportive evidence results in enhancement on the affirmation of one BPA that focuses on some specific elements. Thus, we can introduce a threshold θ of total probability assigned to complementary-set and uncertainty to filter the supportive BPAs and focus on BPAs that assign sufficient probability to specific activity in the inter-classifiers combination to avoid unnecessary calculations when it is necessary.

The holistic combination model can be regarded as a two-layer neural network as depicted in Algorithm 1. On account of the relevance between adjacent time intervals, recent results and current evidence are weighted with additional weight ***W***${}^{P}$ and combined into time domain synthesis evidence called intra-classifier combination. Furthermore, we set n hyper-parameters $\left(N_{i}+1\right)$ where 1≤i≤n to denote the number of time intervals that need to be synthesized for each classifier, in other words, the BPAs of time intervals from $T-N_{i}$ to *T* are involved in each intra-classifier combination to consider the diversity of duration between activities focused by different classifiers and *T* refers to the current time interval. That evidence generated by each classifier in adjacent time intervals is weighted and synthesized into synthetical evidence, and then they are selected to implement another weighted synthesization with ${\hat{W}}^{P}$ called inter-classifiers combination. The following synthesization outputs a consistent probability vector, based on which we can train the model or choose the final activity category. The weighting procedure of classifier *s*${}_{i}$ in time interval *t* is expressed as Equation (7).

(7) $${\left[\begin{array}{c} {\tilde{E}}_{s_{i}a_{1}}^{t}\\ {\tilde{E}}_{s_{i}a_{2}}^{t}\\ \vdots \\ {\tilde{E}}_{s_{i}a_{n}}^{t}\\ {\tilde{E}}_{s_{i}{∁}_{A}a_{i}}^{t}\\ \Omega_{i}^{t} \end{array}\right]}^{T}⊙{\left[\begin{array}{c} W_{i1}^{Pt}\\ W_{i2}^{Pt}\\ \vdots \\ W_{in}^{Pt}\\ W_{in+1}^{Pt}\\ 1 \end{array}\right]}^{T}+{\left[\begin{array}{c} 0\\ 0\\ \vdots \\ 0\\ 0\\ \omega_{i}^{t} \end{array}\right]}^{T}={\left[\begin{array}{c} {\hat{E}}_{s_{i}a_{1}}^{t}\\ {\hat{E}}_{s_{i}a_{2}}^{t}\\ \vdots \\ {\hat{E}}_{s_{i}a_{n}}^{t}\\ {\hat{E}}_{s_{i}{∁}_{A}a_{i}}^{t}\\ {\hat{\Omega }}_{i}^{t} \end{array}\right]}^{T}$$

The size of matrixes in Equation (7) are 1×(n+2). The first matrix ${\tilde{E}}_{s_{i}}^{t}$ denotes the revised evidence of classifier *s*${}_{i}$ in time interval *t* and its element ${\tilde{E}}_{s_{i}a_{j}}$ corresponding to the probability of activity *a*${}_{j}$ has nonzero value if and only if i=j, so as to the element ${\hat{E}}_{s_{i}a_{j}}$ in the weighted evidence that is the last matrix. The first (n+1) elements of the weight matrix has the same value while the last element is a constant 1 for the reason that the last element of the revised evidence matrix denotes the uncertainty and is used to ensure the constancy of the overall probability which is unnecessary to be weighted. ω in the third matrix denotes the uncertainty caused by the additional weights and is added to Ω to obtain the total uncertainty $\hat{\Omega }$. ⊙ represents the Hadamard product and + denotes the common matrix addition. The element ${\hat{\Omega }}_{i}^{t}$ of time interval *t* in the weighted evidence matrix can be written as Equation (8):

(8) $$\begin{array}{cc} {\hat{\Omega }}_{i}^{t} & =\Omega_{i}^{t}+\sum_{j=1}^{n+1}{\tilde{E}}_{s_{i}a_{j}}^{t}\left(1-W_{ij}^{Pt}\right)=\Omega_{i}^{t}+{\tilde{E}}_{s_{i}a_{i}}^{t}\left(1-W_{ii}^{Pt}\right)+{\tilde{E}}_{s_{i}{∁}_{A}a_{i}}^{t}\left(1-W_{in+1}^{Pt}\right)\\ \; & =1-{\tilde{E}}_{s_{i}a_{i}}^{t}W_{ii}^{Pt}-{\tilde{E}}_{s_{i}{∁}_{A}a_{i}}^{t}W_{in+1}^{Pt} \end{array}$$

The weighting process of inter-classifiers combination shares the same procedure with the intra-classifier combination, with only differences in the dimensions of input data and weights. The dimensions of the inter-classifiers combination can be $2^{n}-1$ or n+1 while the latter is n+2 that only have 3 nonzero elements. Both of intra-classifier and inter-classifiers combinations use the basic combination rule described by Equation (8), whereas inputs of the former have the same nonzero elements which results in only numerical variation of the combined BPA while the number of nonzero elements of combined BPA increasing with the amount of input BPAs in the latter. The output of intra-classifier combination corresponding to classifier *s*${}_{i}$ is denoted as ${\hat{E}}_{s_{i}}$ and the final output of inter-classifiers combination is denoted as $E_{f}$.
**Algorithm 1** Time series evidence combination rule**Require:** Revised evidence ${\tilde{E}}_{s_{i}}^{t}$; the hyper-parameters $N_{i}$; the number of classifiers/activities *n*; current time interval *T*;**Ensure:** The final combination result $E_{f}$; 1:Initial the weights $W^{P}$ and ${\hat{W}}^{P}$; 2:Initial $E_{f}=\left[0,0,\cdots ,0,1\right]$; 3:**for**i=1; i≤n; i++**do** 4:    **for** t=T; $t\geq T-N_{i}$; t−− **do** 5:        Calculate each ${\hat{E}}_{s_{i}}^{t}$ of classifier $s_{i}$ in time interval *t* with $W^{P}$ by Equations (7) and (8); 6:    **end for** 7:    Calculate each intra-classifier combination result ${\hat{E}}_{s_{i}}$ of classifier $s_{i}$ by Equation (6); 8:    $E_{f}\leftarrow E_{f}⊕{\hat{W}}^{P}⊙{\hat{E}}_{s_{i}}$ by Equations (6)–(8); 9:**end for**10:**if** train model **then**: Minimize the loss function and calculate the change of weights $\Delta W^{P}$ and $\Delta {\hat{W}}^{P}$; $W^{P}\leftarrow W^{P}+\Delta W^{P}$; ${\hat{W}}^{P}\leftarrow {\hat{W}}^{P}+\Delta {\hat{W}}^{P}$;11:**else if** test model **then**: Output $W^{P}$ and ${\hat{W}}^{P}$;12:**end if**

This combination rule is designed for evidence data and two restrictions are required to ensure the interpretability and reality. The first restriction is the objective limitation of the evidence that all elements are nonnegative and the sum is a constant 1. Moreover, this limitation is worked throughout the whole process and even to the intermediate BPAs that will not be recorded. The other restriction is on the additional weight ***W***${}^{P}$. During the weighting procedure, evidence is rated on the correlation to the final result, and the functional components will multiply with smaller correlation coefficient. Especially, the weight will be the maximum value 1 if and only if the probability assignment of the element is the same with the final outcome. To guarantee the value range of the changing weights, we introduce a new parameter α that has the same size to ***W***${}^{P}$ to represent it, and the numerical relationship between them is defined as Equation (9),

(9) $$W^{P}=\frac{1}{1+e^{-\alpha }}$$

This sigmod function maps the variable that can be any real number to a fixed range from 0 to 1. The loss function used in this model is the cross entropy between the final evidence $E_{f}$ and the expanded label, and the latter is constituted by appending one or $\left(2^{n}-1-n\right)$ additional elements with the value of 0 to the original label to accommodate the occurrence of new element in the output that is corresponding to the uncertainty or other subsets of the universal set. Moreover, a penalty term is added to the loss function to reduce the overfitting risk and increase the rate and stability of the optimization. The regularized cross entropy loss function *L* is expressed as Equation (10),

(10) $$L=-\sum_{j=1}^{n}z_{j}\ln E_{f}^{j}+\frac{\lambda }{2}\sum_{i=1}^{n}\left({\left({\hat{W}}_{i}^{P}\right)}^{2}+\sum_{t=T-N_{i}}^{T}{\left(W_{i}^{Pt}\right)}^{2}\right)$$

where the summation of ***W***${}^{P}t_{i}$ and ${\hat{W}}_{t}^{P}$ is the L2-regularization term, and $\frac{\lambda }{2}$ is the coefficient to adjust the relative magnitude of regularization term and cross entropy. The model is trained with the error back propagation mechanism and gradient descent algorithm, choosing the parameter values that minimize the loss function as the final weights.

## 4. Results

### 4.1. Data Set and Experiment Settings

In this paper, we used an Intel${}^{®}$ Core${}^{\mathrm{TM}}$ I5-7300HQ 2.5GHz CPU (Intel, Santa Clara, CA, USA), two NVIDIA GeForce${}^{®}$ GTX 1080Ti 11GB GPU (NVIDIA, Santa Clara, CA, USA) and Mathworks Matlab${}^{®}$ R2017a (Mathworks, Natick, MA, USA) together with Google${}^{®}$ TensorFlow${}^{\mathrm{TM}}$ 1.4.0 (Google, Mountain View, CA, USA) and open source software Python${}^{\mathrm{TM}}$ 3.5.5 (Python Software Foundation, open source software, Wilmington, DE, USA) to perform the simulations. The main data set is the UCI-HAR data-set [50] which contains 6 activities of 30 volunteers. Those data were acquired by fixing a smart-phone on the waist and 3 channels accelerometer sensor data and 3 channels gyroscope sensor data were recorded simultaneously with a frequency of 50 Hz. The six activities were walking (activity 1), walking up-stairs (activity 2), walking down-stairs (activity 3), sitting (activity 4), standing (activity 5) and lying (activity 6) respectively. Moreover, there were totally 748406 sample points in the raw data, and the input data was converted with the selected window size. The input data was randomly partitioned into two sets, where almost 80% of the data was selected for generating the training data and 20% the test data.

Based on the real time strategy, the data set was divided into small windows of 0.4 s with 50% overlap. 20% of the data was randomly selected as the test data to evaluate the generalization capability of model while 10-fold cross validation method was used on the remaining data to adjust hyper-parameters. The parameters we used in the experiments are shown in Table 1.

### 4.2. Experimental Results

The experiment in analysis of the real time strategy has proved the effectiveness of the small window with a two layer LSTM-based classifier, and further experiments with different sizes of nodes in each hidden layer, which resulted in approximate lengths of windows to reach the maximum evaluation index, has confirmed that the window of 0.4 s contains enough information for simple activity recognition.

**Figure 3:**
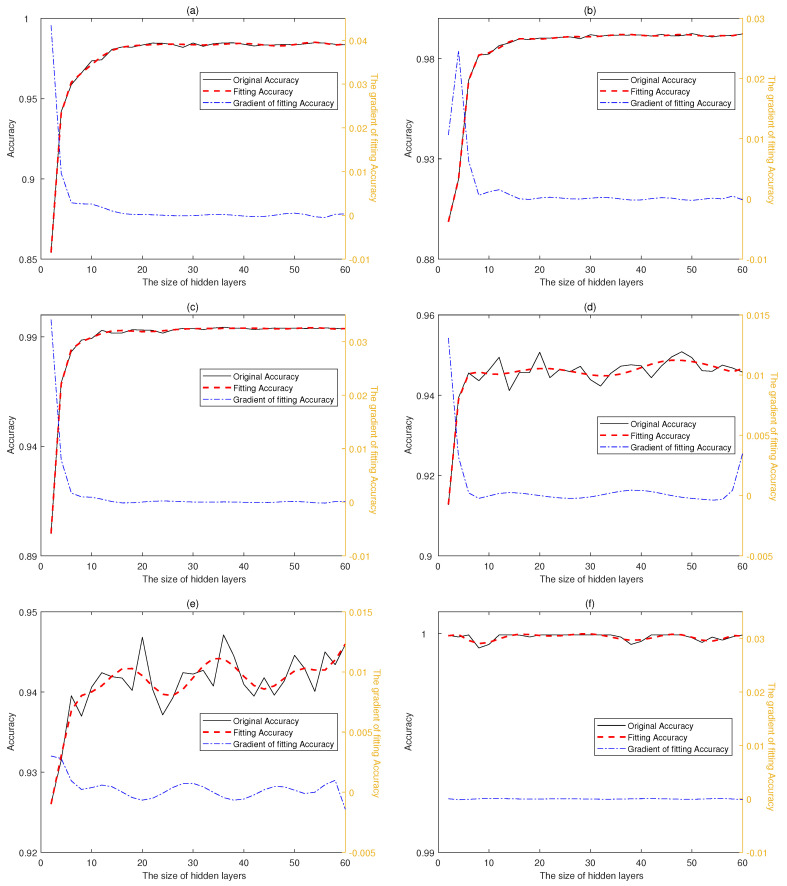
Accuracies of multi-classifiers with different hidden layer sizes. The accuracies variation of classifier 1 (**a**), 2 (**b**), 3 (**c**), 4 (**d**), 5 (**e**), 6 (**f**) corresponding to activity 1, 2, 3, 4, 5, 6 in UCI-HAR data-set.

In this paper, we implement multi-classifiers to recognize activity concurrently. Figure 3 shows the variation of accuracies with different hidden layer sizes, and each point on the graph is an average accuracy on different validation set. There are 3 curves in each figure, the black solid line reflects the change of original accuracy, the red dotted line indicates the fitting accuracy while the last line reflects the gradient of fitting Accuracy. The original accuracy and fitting accuracy share the left y axis while the gradient relies on the right y axis. The accuracies of the first three classifiers increase with the size of hidden layer quickly at the beginning and tend to be stable around 16 while the latter three classifiers fluctuate around different values, which is consistent with the fact that sitting and standing are easy to be confused while laying is much different from other activities. Moreover, the confusion between sitting and standing may be the biggest constraint to the performance of classifiers. Hence, we select [16,16,16] as the final sizes of hidden layers to the first three classifiers to achieve high performance with the lowest consumption of computing resources and [20,20,6] to the latter three classifiers to reach the maximum value of accuracies respectively.

As shown in Table 2, each classifier is corresponding to one activity. Those classifiers are retrained on the train data and tested on the test data to count the evaluation parameters, and then the weighting coefficients of each classifier are calculated by Equations (2) and (3) with the parameters on test data. The evaluation parameters can reflect the performance of classifiers, and we can find that activity 4 and 5 are hard to be distinguished in contrast to other activities. Thus, the corresponding weighting coefficients are smaller than others

**Figure 4:**
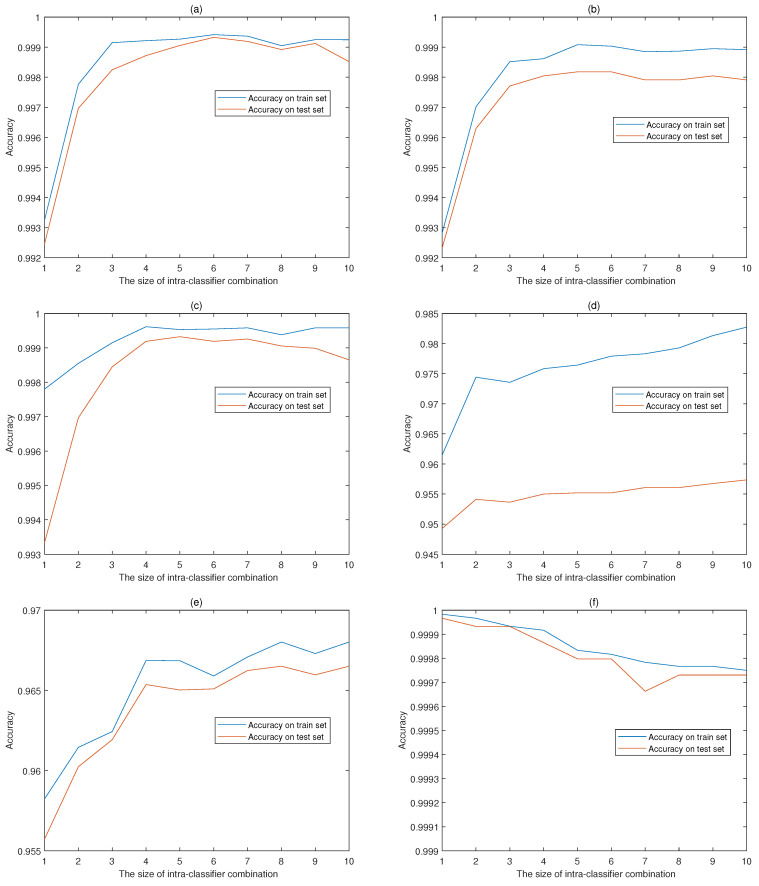
Accuracies of intra-classifier combination with different time window sizes. The intra-classifier combination accuracies of classifier 1 (**a**), 2 (**b**), 3 (**c**), 4 (**d**), 5 (**e**), 6 (**f**) corresponding to activity 1, 2, 3, 4, 5, 6 in UCI-HAR data-set.

Figure 4 shows the accuracies of intra-classifier combination on train data and test data with the size of combination time windows ranging from 1 to 10. There are 2 curves in each figure, the blue solid line reflects the accuracy on train set while the red solid line indicating the accuracy on test set. The accuracies vary in different ranges and the performance on train data generally outperforms the test data, with the exception of classifier 6 which has high accuracies on both data sets. Classifier 1 achieves the maximum accuracy with a time window size of 3 while the other classifiers choose 4, 4, 7, 7 and 1 as the optimal window size respectively. The various window sizes verify the diversity of time correlation among activities.

Then, we compared our model with other human activity recognition methods including classical algorithms, such as PCA and SVM in, and other LSTM-based methods in [51] together with the deep learning neural networks in [42]. As shown in Table 3, the accuracy of using one huge LSTM network with the input window size of 0.4 s reached 93.9% while the result was increasing to 94.9% when the input window size was 1.6 s, and the result was increasing to 95.02% when expanding the hidden layer size to 27. When it comes to parallel subnetworks, the accuracy of choosing the largest output of those subnetworks reached 95.09% while the accuracy was 95.28% when combining the output of subnetworks with inter-classifiers combination algorithm. The accuracy was 96.44% by using the method proposed in this paper while the largest accuracy of the traditional methods was 94.79%. The model proposed in this paper has small advantages in accuracy, but it will be helpful in real-time human activity analysis for its consideration of the tradeoff between model size and time consumption.

Lastly, we designed some control experiments to verify the effectiveness of the strategy of merging complementary set and universal set before the combination process, together with the effectiveness of the intra-classifier combination. Furthermore, we chose the classical Back-Propagation neural network to fuse the evidence generated by P-LSTM-E model to reflect the availability of the evidence combination network. As shown in Table 4, the accuracies of experiments without merger strategy were approximately equal to those with merger strategy, and the accuracies of experiments using the intra-classifier combination strategy were almost 1% larger than those without. Moreover, the combination network proposed in this paper had the same performance as the classical Back-Propagation neural network on the UCI-HAR data-set.

## 5. Discussion

In classification, the information contained in input data and discrimination ability of the model have direct influences on the final result. When it comes to time series data that different categories vary in duration, the window size of input represents the quantity of information, which will be insufficient when the size is too small and will be redundant or incoordinate when the size is too large. In this paper, we attempted a series of window sizes, and the accuracy was increasing with the window size and the growth rate slowed down after a threshold value. Therefore, we chose 0.4 s as the window size based on the tradeoff between accuracy and time cost. However, this small window size resulted in a low accuracy, so we added the intra-classifier combination process to bring in more information, and the time span varied with the categories. The biggest time span in our experiments was 7 windows which meant that the continuous data we used is up to 1.6 s. However, the accuracy of the model proposed in this paper is almost 1% higher than the LSTM model with a window size of 1.6 s.

The P-LSTM-E model adopted n LSTM networks to replace one huge LSTM network where n was the number of categories, the amount of neurons in each hidden layer would be several times than the latter if we treated the former as one network in parallel. However, the amount of connections between different layers neurons was sparse for the reason that the connections was inside the individual networks, which meant that the computation complexity and the number of trainable weights were not based on the amount of neurons of the virtual parallel network. The number of weights in a LSTM network can be calculated by Equation (11).

(11) $$Num=4*\left(\left(N_{in}+N_{h1}\right)*N_{h1}+\left(N_{h1}+N_{h2}\right)*N_{h2}\right)+N_{h2}*N_{out}$$

where *Num*, *N*${}_{in}$, *N*${}_{h1}$, *N*${}_{h2}$ and *N*${}_{out}$ represent the total number of weights, the dimension of input data, the number of neurons in hidden layer 1, the number of neurons in hidden layer 2 and the dimension of output respectively. Based on the parameters used in the model, the total number of weights is 28,188 in P-LSTM-E model which is approximate to 28,674 weights in the huge LSTM network with 27 neurons in each hidden layer. The combination model added additional weights which could be thousands, especially when we chose to extend the dimension to $2^{n}-1$ before inter-classifiers combination. The additional weights was usually less than 300 if we adopted the merger strategy.

The control experiments considered the situation whether to adopted the merger and intra-classifier combination strategies or not. The results showed that combination model adopted merger strategy could achieve approximate accuracies to those without merger strategy, which was happened for the reason that the merger strategy retained enough information and only omitted the details. Meanwhile, the intra-classifier combination strategy bringing in more information was proved to have some improvement on the final accuracy. The combination model substituting combination rules based on evidence theory for common activation functions in neural network was verified to be effective, and this new method has a better explanation of the weighting process and intermediate result in probability theory.

## 6. Conclusions

Activity recognition is closely related to human’s daily life, and it plays an important role in physical rehabilitation and entertainment experience. With the flourishing development of machine learning and artificial intelligence, fine-grained activity recognition with high performance will promote the improvement of personalized service and quality of experience. In this paper, we analyzed the real-time strategy and proposed a LSTM-based evidence generation/combination model. In consideration of the time consumption, the feasibility of small window size strategy has been discussed, and we used multiple parallel LSTM-based classifiers with small network size to construct the generation model, which simplified the classification problem and enhanced the extensibility of model. Then, some evaluation coefficients were presented to revise the results of classifiers. The combination model considered the intra-classifier time domain correlation and the association among multiple classifiers to synthesize revised evidence, which can be regarded as a two-layer neural network while the combinations among nodes are conforming to the combination rule in evidence theory. The results of experiments has proved the effectiveness of the strategy and models, and the final accuracy of the model was over 96.4% with the time window size of only 0.4 s on raw data. Future works will include the analysis of confusable activities, the research on imbalanced data-set and the interaction between image data and wearable sensor data. Likewise, the distance between activities and the encoding method may be the potential research issues.

## Figures and Tables

**Table 1:** Experiment settings.

Parameters	Value
Input window size	1–150
Input data channels	6
LSTM-softmax Classifiers	6
Hidden layer size	(2–62) × 2
Intra-classifier combination size	1–10
Learning rate	0.0015–0.003
Penalty term coefficient	0.0015
Mini-batch size	100–1500
Maximum epochs	200

**Table 2:** Evaluation parameters and weighting coefficients of each classifier.

Classifiers	Train Data				Test Data				Weighting Coefficients	
	TP	FP	TN	FN	TP	FP	TN	FN	$P_{i}$	$P_{n+1}$
1	9651	162	49,985	201	2290	42	12,443	65	0.9554	0.9915
2	9075	142	50,535	247	2279	49	12,441	71	0.9500	0.9904
3	8495	80	51,252	172	2076	45	12,667	52	0.9554	0.9924
4	8404	726	49,222	1647	2153	249	11,974	464	0.7512	0.9438
5	10,564	719	48,138	578	2338	283	11,888	331	0.7920	0.9509
6	10,964	0	49,034	1	2720	0	12,119	1	0.9996	0.9999

**Table 3:** Performance comparison.

Methods	Accuracy	Methods	Accuracy
LSTM + Raw data(0.4 s)	93.9%	PCA+MLP	57.10%
LSTM + Raw data(1.6 s)	94.9%	SVM	89.30%
LSTM + Raw data(hidden layer size 27)	95.02%	Convnet + MLP	94.79%
P-LSTM-E + Maxout	95.09%	Baseline LSTM	90.77%
P-LSTM-E + inter Combination	95.28%	Bi-LSTM	93.79%
P-LSTM-E + (intra and inter) Combination	96.44%		

**Table 4:** Results of control experiments to verify the effectiveness of strategies.

Combination Strategies	7 Hidden Neurons		20 Hidden Neurons	
	Without Intra-Combination	With Intra-Combination	Without Intra-Combination	With Intra-Combination
BPNN	95.31%	96.21%	95.20%	96.33%
Without merger	95.26%	96.40%	95.29%	96.49%
With merger	95.28%	96.46%	95.34%	96.48%
